# Sudden cardiac death in Africa

**Published:** 2014

**Authors:** Ashley Chin

**Affiliations:** Cardiac Clinic, Groote Schuur Hospital, University of Cape Town, Cape Town, South Africa

Africa is facing a huge health burden of both communicable and non-communicable diseases. Cardiovascular disease is becoming an important cause of mortality in Africa.^1^ Unfortunately, statistics on the incidence of cardiovascular disease in Africa are not readily available and in many sub-Saharan African countries there is no information or only poor-quality data.

Sudden cardiac death (SCD) is an unexpected natural death due to cardiovascular disease that occurs within one hour of the onset of symptoms.^2^ The epidemiology of SCD in Africa is unknown. In the United States, SCD results in approximately 300 000 deaths per year.^2^ Ischaemic heart disease is responsible for 80% of cases, followed by non-ischaemic myopathic diseases, such as hypertrophic and dilated cardiomyopathy, which account for 10 to 15% of cases. Approximately 5% of cases of SCD can be attributed to primary electrical disorders such as congenital long QT syndrome and Brugada syndrome.

In this article of the Journal, Bonny *et al*. and the Pan-African Society of Cardiology task force on Sudden Cardiac Death present the rationale and design of the Pan-African SCD study (page 176). This will be a multicentre, community-based, prospective cohort registry that will report on cases of SCD from all African regions. The well-designed, epidemiological study will be the first and largest of its kind from Africa. This registry will be community-based and led by senior physicians, working together with a multidisciplinary team. Genetic testing and autopsy data will be obtained to confirm clinical diagnoses as far as possible.

This study is important for several reasons. The Pan-African SCD study may highlight important differences in the incidence and relative causes of SCD in Africa.

In contrast to the developed world, cardiovascular disease in Africa is still predominantly non-ischaemic.^1^,^3^ The THESUSHF registry has confirmed that the major causes of acute heart failure in Africa are non-ischaemic. Hypertensive heart disease, cardiomyopathy (peripartum cardiomyopathy, idiopathic dilated cardiomyopathy and endomyocardial fibrosis), pericardial disease and rheumatic valvular heart disease account for the majority of cases. Left ventricular hypertrophy, whether confirmed by ECG or echocardiogram, is a strong independent predictor of cardiovascular death and SCD.^2^ An autopsy study from Nigeria found that hypertensive heart disease was the most common cause of SCD in that region.^4^ Therefore the Pan-African SCD study may provide insight into the burden of SCD due to neglected diseases that are endemic to Africa, such as rheumatic valvular heart disease and tuberculous myo/pericardial disease.

Tuberculosis is a major cause of mortality in Africa and reports suggest that tuberculous myocarditis may cause SCD.^1^,^5^ While SCD due to degenerative aortic stenosis and mitral regurgitation is well appreciated, less is known about SCD in patients with underlying rheumatic aortic and mitral valve disease. These pre-transitional diseases are now uncommon in the developed world and have been neglected in major society guidelines of implantable cardioverter defibrillators (ICDs) in the prevention and treatment of SCD.^6^ This study has important implications for other developing countries, such as India and China, where these diseases are still prevalent.

This study will also highlight the magnitude of well-recognised causes of SCD in Africa. Countries in Africa find themselves at different stages of the epidemiological transition. Many countries, such as South Africa, have reported a rising incidence of ischaemic heart disease.^7^ According to the Global Burden of Disease in 2010, ischaemic heart disease was responsible for more deaths in Africa than either rheumatic heart disease or hypertensive heart disease.^1^

With the introduction of antiretroviral therapy (ART), patients with HIV/AIDS are living longer, and with the increasing atherogenic complications of ART, sub-Saharan Africa is facing an impending epidemic of cardiovascular and metabolic disease.^8^ The Pan-African SCD study will provide contemporary data on the burden of SCD secondary to ventricular arrhythmias related to ischaemic heart disease.

There is little doubt that the primary inherited arrhythmia syndromes [congenital long QT syndrome, arrhythmogenic right ventricular dysplasia (ARVD), Brugada syndrome, early repolarisation (ER) syndrome] exist in Africa. These diseases are frequently under-recognised and are missed diagnoses. Nevertheless, there have been several large cohorts reported from Africa, which report a similar presentation to other populations around the world.

In a large series of 50 ARVD patients from South Africa, the study found a similar clinical presentation and an annual mortality rate comparable to other large registries in North America and Europe.^9^ Brugada syndrome has also been described in 23 patients from Tunisia, who share a similar clinical profile to their Asiatic and Western counterparts.^10^ In another series of 44 congenital long QT 1 syndrome patients in South Africa, a strong founder effect was found, with a single mutation responsible for 52% of cases.^11^ An important study finding will be whether the ER pattern is associated with SCD in young black Africans.

While a report from Cameroon reported that the ER pattern may occur in up to 20% of patients who present with cardiovascular symptoms, a recent study found that the ER pattern in the precordial leads was not associated with increased mortality in black African-Americans.^12^,^13^ If an association of the ER pattern and SCD indeed exists, it will have major clinical implications because of the high prevalence rate of this ECG pattern in black Africans.

The study will face significant challenges in terms of diagnosis and management. First, one can expect that a significant proportion of patients who survive SCD will be managed by general physicians or cardiologists, who may have limited experience in the diagnosis and management of these complex patients.

Second, special investigations such as echocardiography, cardiac catheterisation, magnetic resonance imaging, electrophysiological testing and Holter monitoring may be unavailable in many African centres. Certain diseases, such as ARVD, which require multiple investigations to make a diagnosis, may prove difficult to diagnose in the absence of clinical expertise and special investigations.

Third, from a therapeutic standpoint, treatment options will be limited in many countries for financial reasons. Will ICDs be available for secondary prevention and are there enough skilled doctors to implant them? Hospitals and healthcare systems will face huge financial challenges in funding these high-cost devices, even in the secondary-prevention setting. A paucity of cardiologists and electrophysiologists exists in sub-Saharan and west Africa with very few or no centres able to implant pacemakers and ICDs. Even in north African countries, where the situation is slightly better, ICD centres are few.^14^

This study has the potential to create a legacy on the management of SCD in Africa. Regional centres participating in this study have the potential to become centres of expertise in the management of SCD. Information gained from this study may help governments develop healthcare policies, including the placement of defibrillators in public places, such as schools, sports venues and health facilities, to improve out-of-hospital resuscitation attempts; and providing adequate funding for high-cost devices.

Recognising the paucity of expertise may facilitate training of future African physicians and cardiologists. Determining the magnitude and nature of the problem of SCD in Africa, which is the main goal of this study, is the first major step.
